# Prediction model for patients with acute respiratory distress syndrome: use of a genetic algorithm to develop a neural network model

**DOI:** 10.7717/peerj.7719

**Published:** 2019-09-16

**Authors:** Zhongheng Zhang

**Affiliations:** Department of Emergency Medicine, Sir Run Run Shaw Hospital, Zhejiang University School of Medicine, Hangzhou, China

**Keywords:** Acute respiratory distress syndrome, Prediction, Neural networks, Mortality, Genomic algorithms, Genetic algorithm

## Abstract

**Background:**

Acute respiratory distress syndrome (ARDS) is associated with significantly increased risk of death, and early risk stratification may help to choose the appropriate treatment. The study aimed to develop a neural network model by using a genetic algorithm (GA) for the prediction of mortality in patients with ARDS.

**Methods:**

This was a secondary analysis of two multicenter randomized controlled trials conducted in forty-four hospitals that are members of the National Heart, Lung, and Blood Institute, founded to create an acute respiratory distress syndrome Clinical Trials Network. Model training and validation were performed using the SAILS and OMEGA studies, respectively. A GA was employed to screen variables in order to predict 90-day mortality, and a neural network model was trained for the prediction. This machine learning model was compared to the logistic regression model and APACHE III score in the validation cohort.

**Results:**

A total number of 1,071 ARDS patients were included for analysis. The GA search identified seven important variables, which were age, AIDS, leukemia, metastatic tumor, hepatic failure, lowest albumin, and FiO_2_. A representative neural network model was constructed using the forward selection procedure. The area under the curve (AUC) of the neural network model evaluated with the validation cohort was 0.821 (95% CI [0.753–0.888]), which was greater than the APACHE III score (0.665; 95% CI [0.590–0.739]; *p* = 0.002 by Delong’s test) and logistic regression model, albeit not statistically significant (0.743; 95% CI [0.669–0.817], *p* = 0.130 by Delong’s test).

**Conclusions:**

The study developed a neural network model using a GA, which outperformed conventional scoring systems for the prediction of mortality in ARDS patients.

## Background

Patients with acute respiratory distress syndrome (ARDS) are at increased risk of death. If the underlying disease is not well treated, mild ARDS may progress into a more severe form, where admission to an intensive care unit (ICU) is required (Umbrello et al., 2016). Additionally, patients at this stage may require mechanical ventilation to avert life-threatening hypoxia (Abdel Hakim et al., 2016). A variety of mechanical ventilation strategies, such as low tidal volume ventilation, prone positioning and paralytics have been developed over the past few decades in order to improve clinical outcomes of ARDS (Carron, 2016). However, the improvement in mortality rate was less than satisfactory (Zhang, Chen & Ni, 2015; Mezidi & Guérin, 2016), and there is still much work to be done in this area. Risk stratification for ARDS can be a useful tool in medical decision making and the design of clinical trials, thus strenuous efforts have been made to derive a model for the prediction of ARDS mortality (Cooke et al., 2009; Frenzel et al., 2011; Balzer et al., 2016; Zhao et al., 2017). The Acute Physiology and Chronic Health Evaluation (APACHE) III score is a severity-of-disease classification system, which is applied within the first 24 h of admission to an ICU, higher scores correspond to more severe disease forms and a higher risk of mortality. For decades, APACHE III has been widely used for the prediction of ARDS mortality (Knaus et al., 1991). However, most of these studies employed conventional regression methods to develop prediction models, which requires preexisting domain knowledge for model interactions and/or higher-order terms; while sophisticated machine learning methods can capture these complex relationships automatically based on the data.

A genetic algorithm (GA) is an adaptive heuristic search algorithm based on the evolutionary ideas of natural selection and genetics. As such it represents an intelligent exploitation of a random search used to solve optimization problems (Lucasius & Kateman, 1993). Variable selection in building prediction models is a problem of optimization. A GA is suitable for large-scale searches of candidate predictors, and it is a popular method in many fields such as chemistry, computer science and economics (Lucasius & Kateman, 1993; Las Heras et al., 2016; Escalona-Vargas et al., 2016). However, GAs have not yet been widely used in clinical research, mainly due to their complexity in computations. In the present study, I aimed to develop a neural network model for the prediction of ARDS mortality, with predictor selection being performed using a GA. The final model was compared to the model developed using a conventional logistic regression approach and the existing risk prediction score APACHE III.

## Materials and Methods

### Training and validation cohorts

The study was a secondary analysis of two randomized controlled trials (RCTs) involving ARDS. The Statins for Acutely Injured Lungs from Sepsis (SAILS, NCT00979121) study enrolled 745 patients with sepsis-induced ARDS. Patients were randomized to receive either rosuvastatin or a placebo in a double-blind manner. The result was neutral in that rosuvastatin was not able to reduce mortality in comparison to the placebo (Truwit et al., 2014). The other study was the OMEGA study (NCT00609180), which enrolled 272 adults within 48 h of developing ARDS (Rice et al., 2011). The OMEGA study also failed to identify beneficial effects of the intervention. The SAILS trial was used for model development and the OMEGA trial was used for model validation. All the data were de-identified and were openly accessible from the Biologic Specimen and Data Repository Information Coordinating Center (BioLINCC, https://biolincc.nhlbi.nih.gov/home/). The study was approved by the ethics committee of Sir Run-Run Shaw Hospital (approval number: 20170313-2) and was performed in accordance with the Declaration of Helsinki.

### Descriptive statistics

The variables included for analysis were compared between survivors and non-survivors. Normally distributed numeric variables were expressed as mean and standard deviation, and they were compared using Student’s *t*-test. Otherwise, they were described as median and interquartile range, and compared using Mann–Whitney U tests. Categorical data were expressed as numbers and percentages, and the differences were compared using Chi-square test or Fisher’s exact test as appropriate. A two-tailed *p* value <0.05 was considered to be statistically significant.

### Variables included for GA

The primary outcome of the study was 90-day mortality, which was coded for if the patient died prior to discharge with unassisted breathing or died prior to achieving unassisted breathing at home for 48 h.

All variables collected during the 24 h before randomization in the original RCTs were included for the GA search. A total of 88 variables were included, which contained information on demographics, admission resources, admission type, laboratory findings, vital signs, parameters of mechanical ventilation, and the outcome status during the study period (Table S1). Hospital admission type referred to the category of hospital admission. Admission sources referred to the location where the patient was immediately prior to the ICU admission, including operating room (OR), recovery room, emergency room (ER), hospital floor, another special care unit, another hospital, direct admit, and step-down unit. The place of residence was the place of residence prior to the admission to hospital. Chronic health information was updated at any time during the admission, which included: acquired immunodeficiency syndrome (AIDS), leukemia (e.g., including acute myeloid leukemia, chronic myeloid leukemia, all lymphocytic leukemia, and multiple myeloma), Non-Hodgkin’s Lymphoma, solid tumor with metastasis, immune suppression (e.g., the patient is immunocompromised secondary to chemotherapy, radiation therapy, use of anti-rejection drugs taken after organ transplant, or the daily use of high doses of steroids (0.3 mg prednisone kg/day or equivalent therapy) within part of or the entire previous six months), hepatic failure (e.g., the patient has decompensated cirrhosis, as evidenced by one or more episodes of jaundice and ascites, upper gastrointestinal bleeding or hepatic encephalopathy or comas), and dementia. Ventilator variables included minute ventilation volume measured as the total tidal volume summed over one minute. Physiological variables included temperature, systolic blood pressure, mean arterial pressure, heart rate, respiratory rate, and urine output. Laboratory variables included hematocrit, white blood cell count, platelet count, serum sodium, potassium, creatinine, albumin and bicarbonate. All variables were obtained 24 h preceding randomization. In the case where there were several measurements of one variable, the ones associated with the worst illness severity was used, for example, both lowest and highest body temperatures were included for analysis because both low and high temperatures were associated with increased risk of mortality as compared with the normal temperature. Intraoperative values or values related to death or cardiac arrest situations were not included for analysis. If no values were obtained for clinical purposes during the 24 h preceding randomization, the laboratory tests were obtained after obtaining patient/surrogate consent, but before initiating study procedures. The ventilator parameters were obtained on day 0. The delivered tidal volume was calculated as the inspired tidal volume (ml) set on the ventilator, minus any additional tidal volume added to correct for compression and ventilator tube expansion (note that Puritan-Bennett 7,200’s and some other ventilators make this correction automatically). The plateau pressure measurement should be made with a 0.5-second inspiratory pause. Peak inspiratory pressure was obtained while the patient was relaxed, not coughing or moving in bed. Continuous variables were included in their original forms. Categorical variables were converted to dummy variables. Variables with >10% missing values were excluded, however variables (bilirubin, albumin, glucose, sodium and inspiratory oxygen fraction) with <10% missing values were compensated for using a single imputation (the *mice* package version 3.3.0) (Van Buuren & Groothuis-Oudshoorn, 2011).

**Figure 1:**
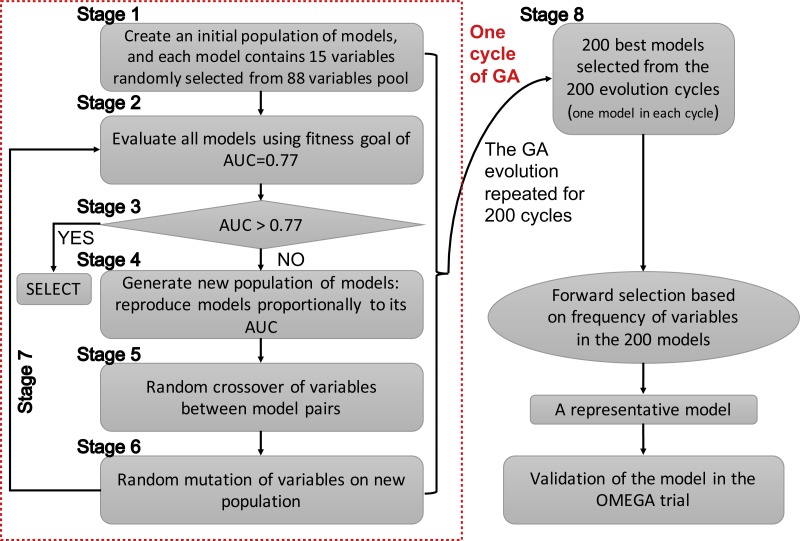
Schematic representation of the genetic algorithm.

Since GAs were originally developed for the selection of genes, the terms “gene” and “chromosome” were widely used in the field of bioinformatics. However, the use of these terms in this manner might be confusing in the present study. Herein, I clarify that the GA searching algorithm was employed to search important clinical variables, which were related to mortality, one clinical variable was regarded as a “gene” and a group of clinical variables (genes) was regarded as a “chromosome”. The chromosome size was 15 in the search for candidate predictors. The whole process of GA evolution is shown in Fig. 1. A neural network with one hidden layer of six units was used as the classification method. The terms evolution epochs and chromosomes refer to different things. In one evolution epoch, there can be hundreds of models being developed to form the chromosome pool, and I select the one with the best fitness value. A maximum solution of 200 evolutions was used, indicating that a total of 200 independent evolutions/cycles would take place. Studies had reported that the area under the curve for ARDS mortality prediction ranged between 0.67–0.74 (Damluji et al., 2011; Klinzing et al., 2015; Zhao et al., 2017). Since I hypothesized that the prediction accuracy could be better by using GA search, the fitness goal was set to be 0.77. Furthermore, the fitness goal was chosen so that most evolution epochs can reach the goal, but not too quickly with a small number of epochs/generations (e.g., a number of 200 epochs was used in the study). The area under the curve (AUC) fitness goal was set by trying several iterations. The GA search was performed in the training dataset. The study employed the GALGO package (version 1.4) in R to perform GA search (Trevino & Falciani, 2006).

### Developing a representative model

The initial search identified 200 chromosomes that were the best ones in their respective evolution cycles (e.g., a total of 200 GA evolution cycles were performed, and each cycle resulted in one best-fit model with AUC >0.77, if the fitness goal of 0.77 was reached). Although these models all reached the fitness goal, it was not clear which one should be chosen for developing a classifier. Thus, it was reasonable to develop a representative model. The frequency of genes in the population of chromosomes was used as a criterion for inclusion in a pre-selection procedure. I would choose a model with the smallest number of covariates, as long as it is within 99% of the maximum fitness value. Other alternative models with high classification accuracy would also be scored in the *Galgo* object for reference.

### Logistic regression modelling

A logistic regression model was built to compare it to the neural network model developed by GA. The logistic regression model was trained with the SAILS trial. Variable selection was performed by using a stepwise approach with forward selection and backward elimination methods. The model was chosen by Akaike information criterion (AIC), with lower values indicating a better model. The MASS package (version 7.3–50) was employed for the analysis (Venables & Ripley, 2002).

## Model Validation and Comparison with Other Prediction Models

Subjects from the OMEGA trial were used for model validation. The AUC of the model was computed to show the diagnostic performance of the model. Furthermore, I compared the neural network model with the APACHE III score and the model developed by stepwise development of a logistic regression model. The APACHE III score was used because it was a widely used prediction score for unselected ICU patients (Knaus et al., 1991). I hypothesized that our model (i.e., the GA/NN model) would be better than the APACHE III score. Since the logistic regression model was the most widely used statistical tool in predictive analytics in clinical research, the GA model was also compared with the logistic regression model. The DeLong method was used to compare the difference between two receiver operating characteristic (ROC) curves (DeLong, DeLong & Clarke-Pearson, 1988; Robin et al., 2011). A two-tailed *p* value less than 0.05 was considered to be statistically significant.

## Results

### GA search

**Figure 2:**
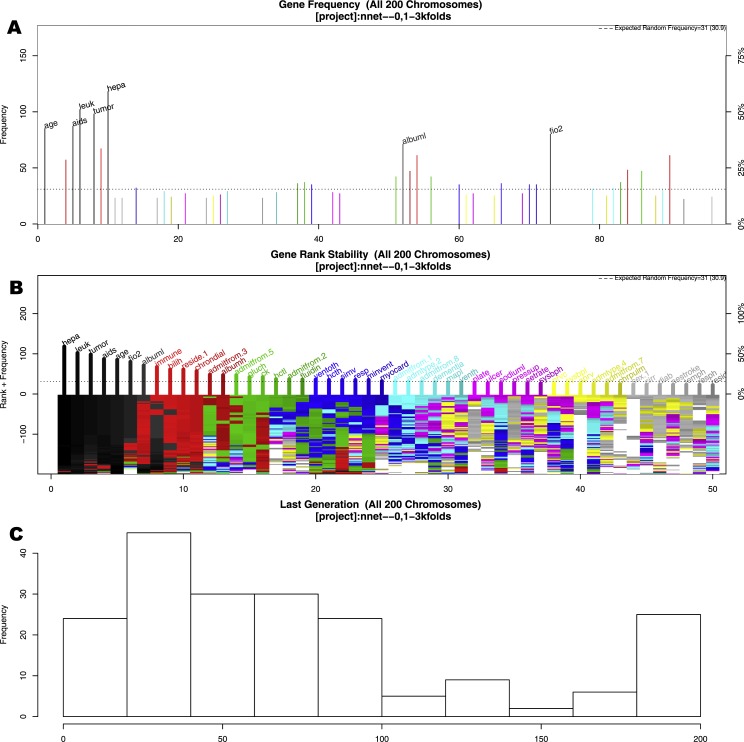
Results of genetic algorithm search. (A) shows the frequency of each “gene” (clinical variable) presented in stored “chromosomes” (a combination of clinical variables). The top 50 variables were colored, and the top seven variables were named. (B) displays the stability of the rank of the top 50 variables. It appeared that the top four variables stabilized quicker. (C) shows the distribution of the number of generations required for an evolution to achieve the fitness goal. If an evolution epoch cannot reach the fitness goal of AUC = 0.77, the iteration is considered as “no solution” and the current iteration stopped. The training sample was split into the training and test sets in 2:1 ratio. Annotations: aids: acquired immunodeficiency syndrome; tumor: metastatic tumor; leuk: leukemia; hepa: hepatic failure; bilih: highest bilirubin; fio2: Fraction of Inspired Oxygen; albuml: lowest albumin; immune: immunodeficiency; hcth: highest value of hematocrit; reside: residence prior to admission; admitfrom: admission source; gluch: highest glucose; pip: peak inspiratory pressure on day 0; resp: respiratory rate on day 0; sodiumh: highest sodium value.

The GA search identified seven important variables associated with mortality (Fig. 2). These variables were age, AIDS, leukemia, metastatic tumor, hepatic failure, lowest albumin, and FiO2. Figure 2A shows the frequency of each variable (gene) presented in the stored chromosomes. The top 50 variables were colored, and the top seven variables were named. Figure 2B displays the stability of the rank of the top 50 variables. It appears that the top four variables stabilized quickly. The red colored variables such as albumin, immunodeficiency, residence prior to admission and chronic dialysis stabilized after approximately 100 epochs/generations. At the right side of Fig. 2B, variables had many changes in ranks (e.g., there were different colors under their names). These variables were considered to be unstable. Perhaps the low ranked “gray” variables require thousands of evolutions to be stabilized. Since they were not important for mortality prediction, we did not run thousands of cycles for them to be stabilized. Figure 2C shows the distribution of the number of generations required for an evolution to achieve the fitness goal.

### Developing a representative model

**Figure 3:**
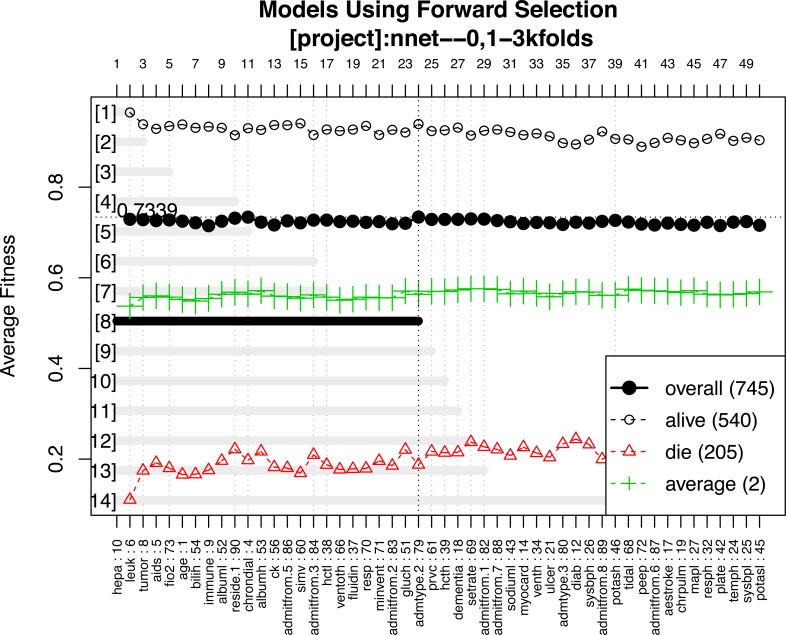
Forward selection using the most frequent variables. Horizontal axis represents the variables ordered by their rank. Vertical axis shows the classification accuracy. Solid line represents the overall misclassification (misclassified samples divided by the total number of samples). Colored dashed lines represent the accuracy per class. One model resulted from the selection whose fitness value is maximum (black thick line), but 9 models were finally reported because they were very similar in absolute value. Annotation: hepa: hepatic failure; leuk: leukemia; tumor: metastatic tumor; aids: acquired immunodeficiency syndrome; fio2: Fraction of Inspired Oxygen; bilih: highest bilirubin; immune: immunodeficiency; albuml: low albumin; chrondial: chronic dialysis; albumh: high albumin; ck: creatinine kinase; simv: simultaneous intermittent mechanical ventilation; hctl: lowest value of hematocrit; reside: residence prior to admission; admitfrom: admission source; ventoth: other ventilation mode; fluidin: fluid intake; gluch: highest glucose; minvent: minute ventilation volume on day 0; resp: respiratory rate on day 0; admtype: admission type.

**Figure 4:**
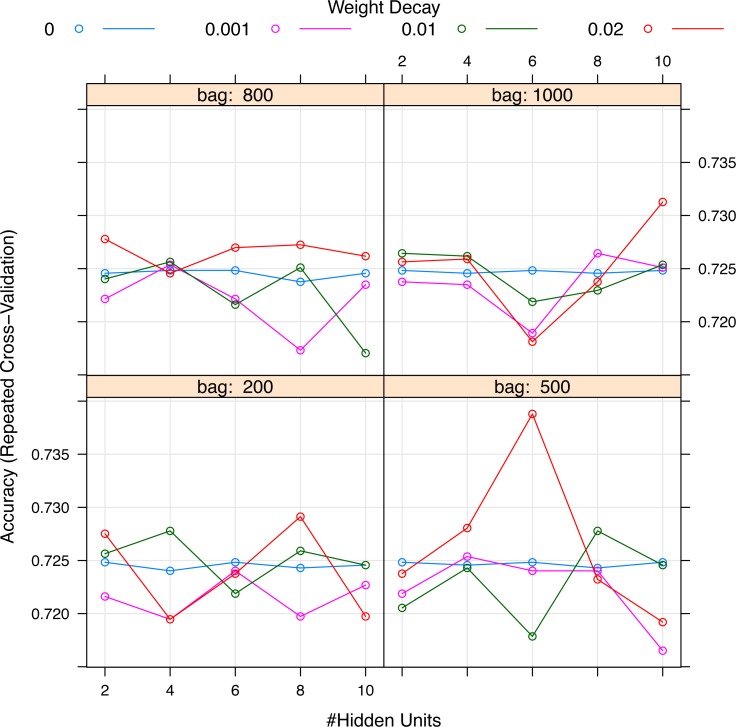
Grid search for hyperparameters of neural networks model. The hyperparameters included the number of hidden units, the number of bags and decay weight.

A representative model was selected by using the forward selection method (Fig. 3). The criteria to choose a model was that the model consisted of the smallest number of covariates, as long as its fitness value was within 99% of the maximum fitness value. The selection was done by evaluating the test error using the fitness function in all test sets. The figure shows 14 models with the best predictive accuracy. The model labelled 8, containing the 24 most frequent variables, was the best model in terms of accuracy. The other 13 models displayed in the figure are within 99% of the maximum fitness value. Model 8 included variables such as immunodeficiency, metastatic tumor, hepatic failure, residing at home independently, FiO2, chronic dialysis, ventilation mode, albumin, age, highest glucose, highest bilirubin, minute ventilation volume (i.e., the product of tidal volume multiplied by respiratory rate) and admission source (i.e., the location where the patient was immediately prior to ICU admission), showed the highest fitness value and was selected as the representative model. The neural network model was trained with these variables. The hyperparameter tuning is shown in Fig. 4.

**Figure 5:**
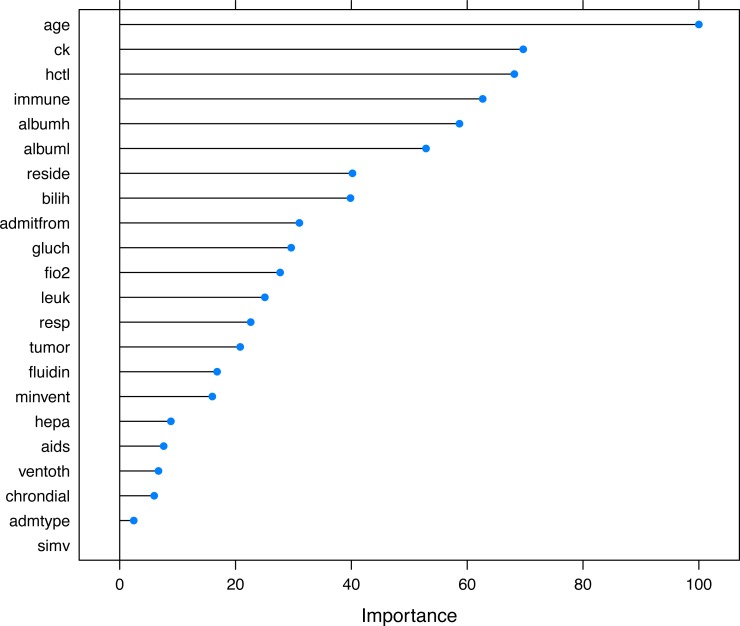
Variable importance in the neural networks model. Variable importance describes the relative importance of explanatory variables for a single response variable in a supervised neural network by deconstructing the model weights. The relative importance (or strength of association) of a specific explanatory variable for the response variable, can be determined by identifying all weighted connections between the nodes of interest. Annotations: hepa: hepatic failure; leuk: leukemia; tumor: metastatic tumor; aids: acquired immunodeficiency syndrome; fio2: inspiratory oxygen concentration; bilih: highest bilirubin; immune: immunodeficiency; albuml: low albumin; chrondial: chronic dialysis; albumh: high albumin; ck: creatinine kinase; simv: simultaneous intermittent mechanical ventilation; hctl: lowest value of hematocrit; reside: residence prior to admission; admitfrom: admission source; ventoth: other ventilation mode; fluidin: fluid intake; gluch: highest glucose; minvent: minute ventilation volume on day 0; resp: respiratory rate on day 0; admtype: admission type.

The selected variables were compared between survivors and non-survivors by univariable analysis in Table 1. The results showed that most of these variables were significantly different between survivors and non-survivors (*p* < 0.05). Figure 5 shows the importance of the variables in the neural network model, which showed that age was the most important variable, followed by creatinine kinase, hematocrit and so on. Table 2 shows the result of the logistic regression model, which showed that most variables were independently associated with mortality.

**Table 1:** Univariate comparison between survivors and non-survivors in the study population.

	Survivor (*n* = 753)	Non-survivor (*n* = 264)	*p*
Immunodeficiency, *n* (%)	76 (10.1)	67 (25.4)	<0.001^*^
Metastatic tumor, *n* (%)	18 (2.4)	22 (8.3)	<0.001^*^
Hepatic failure, *n* (%)	4 (0.5)	8 (3.0)	0.004^*^
AIDS, *n* (%)	15 (2.0)	13 (4.9)	0.022^*^
Residence prior to admission, *n* (%)			0.006^*^
Home independently	625 (83.0)	189 (71.9)	
Home with help	68 (9.0)	39 (14.8)	
Home with professional help (nursing/nursing service)	10 (1.3)	4 (1.5)	
Intermediate care or rehab facility	12 (1.6)	9 (3.4)	
Skilled nursing facility	28 (3.7)	17 (6.5)	
Others	10 (1.3)	5 (1.9)	
Lowest albumin (mg/dl), mean (SD)	2.26 (0.63)	2.10 (0.66)	0.001^**^
Admission source, *n* (%)			<0.001^*^
OR	26 (3.5)	6 (2.3)	
Recovery room	13 (1.7)	0 (0.0)	
ER	324 (43.0)	91 (34.5)	
Floor	177 (23.5)	94 (35.6)	
Another special care unit	12 (1.6)	10 (3.8)	
Another hospital	158 (21.0)	44 (16.7)	
Direct admit	10 (1.3)	6 (2.3)	
Stepdown unit	33 (4.4)	13 (4.9)	
Lowest hematocrit (%), mean (SD)	30.20 (5.98)	28.81 (6.27)	0.001^**^
Highest glucose (mmol/l), median (25th, 75th percentiles)	143.00 [116.00, 182.00]	154.50 [113.75, 204.00]	0.072^***^
Leukemia, *n* (%)	27 (3.6)	26 (9.8)	<0.001^*^
Age (years), mean (SD)	52.00 (15.87)	60.38 (16.59)	<0.001^**^
Highest bilirubin (mg/dl), median (25th, 75th percentiles)	0.80 [0.50, 1.30]	0.90 [0.57, 1.83]	0.004^***^
Highest albumin (mg/dl), mean (SD)	2.36 (0.70)	2.16 (0.68)	<0.001^**^
Chronic dialysis, *n* (%)	11 (1.5)	14 (5.3)	0.001^*^
Admission type, *n* (%)			0.961^*^
Medical	667 (88.6)	234 (88.6)	
Surgical scheduled	23 (3.1)	8 (3.0)	
Surgical unscheduled	52 (6.9)	17 (6.4)	
Other	11 (1.5)	5 (1.9)	

**Notes.**

Abbreviations OR operating room ER emergency room SD standard deviation IQR interquartile range

* Chi-squared test.

** Student’s *t*-test.

*** Mann–Whitney *U* test.

**Table 2:** Logistic regression model for prediction of ARDS mortality.

Variables	OR [95% CI]	*p*
Age (with each year increase)	1.04 [1.03, 1.06]	<0.001
Sex (male as reference)	0.62 [0.41, 0.92]	0.019
AIDS	4.77 [1.52, 14.90]	0.007
Immunodeficiency	3.80 [2.30, 6.32]	<0.001
Hepatic failure	4.07 [0.81, 25.34]	0.102
Cirrhosis	3.47 [1.42, 8.34]	0.006
Prior stroke with sequelae	0.31 [0.07, 1.08]	0.082
Dementia	4.07 [1.55, 11.03]	0.005
Use of vasopressor	1.62 [1.04, 2.56]	0.035
Highest temperature	0.83 [0.67, 1.04]	0.103
Lowest mean arterial BP	0.96 [0.94, 0.99]	0.003
Lowest heart rate	1.02 [1.00, 1.03]	0.012
Mechanical ventilation	2.14 [1.15, 4.14]	0.020
Highest albumin	0.74 [0.54, 1.01]	0.058
Lowest bicarbonate	1.05 [1.01, 1.09]	0.022
Pressure release volume control	0.26 [0.13, 0.52]	<0.001
Pressure support	0.36 [0.16, 0.78]	0.011
Volume assisted	0.26 [0.14, 0.50]	<0.001
Pressure control inverse ratio ventilation	0.06 [0.00, 0.64]	0.037
Airway pressure release ventilation	0.02 [0.00, 0.15]	0.001
Ventilation of other type	0.07 [0.01, 0.28]	0.001
High frequency oscillation ventilation	0.22 [0.02, 1.60]	0.159
Respiratory rate	1.03 [1.00, 1.07]	0.046
PEEP	0.84 [0.77, 0.92]	<0.001
FiO2	4.60 [1.27, 16.75]	0.020
Plateau pressure	1.08 [1.03, 1.14]	0.002
Peak inspiratory pressure	0.96 [0.93, 0.99]	0.020
Mean airway pressure	1.08 [0.99, 1.18]	0.075
Platelet (with each 50-unit increase)	0.91 [0.84, 0.99]	0.037
Lowest systolic BP (with each 20-mmHg increase)	2.00 [1.36, 2.97]	0.001
Highest systolic BP (with each 20-mmHg increase)	0.79 [0.66, 0.93]	0.007
C-reactive protein (with each 20-mg/dl increase)	1.11 [0.97, 1.27]	0.131
Alanine Aminotransferase (with each 10-unit increase)	0.93 [0.85, 1.02]	0.139
Aspartate Aminotransferase (with each 10-unit increase)	1.06 [1.00, 1.13]	0.068
Highest glucose (with each 50-unit increase)	1.22 [1.10, 1.36]	<0.001

**Notes.**

Abbreviations AIDS acquired immunodeficiency syndrome FiO2 fraction of inspired oxygen PEEP positive airway pressure OR odds ratio CI confidence interval

OR was reported for each 1 unit increase for continuous variables if not specified.

**Table 3:** Comparisons of the three models in the testing dataset.

Models	AUC [95% CI]	Sensitivity [95% CI]	Specificity [95% CI]
Neural networks model	0.821 [0.753, 0.888]	0.800 [0.667, 0.917]	0.731 [0.613, 0.896]
Logistic regression model	0.743 [0.669, 0.817]	0.763 [0.644, 0.864]	0.681 [0.474, 0.826]
APACHE III	0.665 [0.590, 0.739]	0.742 [0.583, 0.879]	0.609 [0.446, 0.707]

**Notes.**

Abbreviations AUC area under the receiver operating characteristic curve CI confidence interval APACHE Acute Physiology and Chronic Health Evaluation

The AUC value of neural networks model was not significantly greater than the logistic regression model (*p* = 0.130 by Delong’s test) but was significantly greater the APACHE III score (*p* = 0.002 by Delong’s test).

### External validation of the model

**Figure 6:**
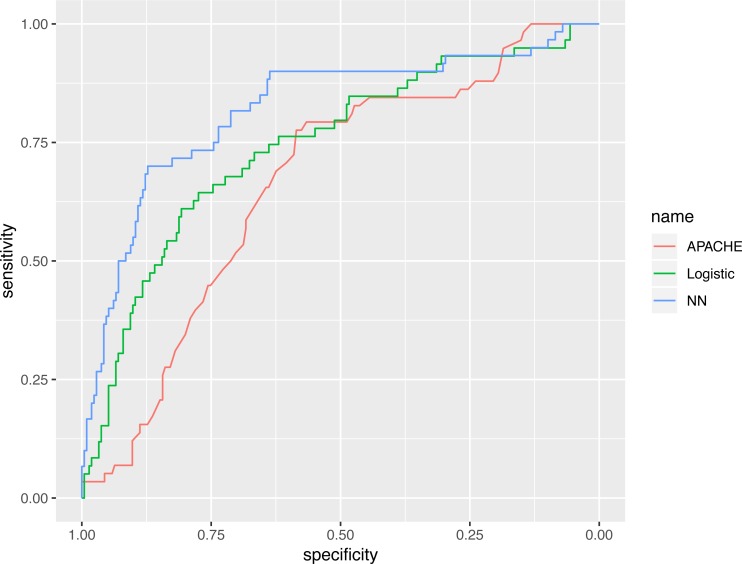
Receiver operating characteristics curves for the three models. The AUC value of neural networks model was not significantly greater than the logistic regression model (*p* = 0.130 by Delong’s test), however it was significantly greater than the APACHE III score (*p* = 0.002 by Delong’s test).

The representative model was developed by finding the best fit to a neural network model, using variables selected by the GA. The neural network model was compared with various predictive scores (Table 3). The AUC of the neural network model, evaluated in the validation cohort, was 0.821 (95% CI [0.753–0.888]), which was numerically greater than the APACHE III (0.665; 95% CI [0.590–0.739]) and the logistic regression model (0.743; 95% CI [0.669–0.817]). The AUC value of the neural networks model, using statistical testing, was not significantly greater than the logistic regression model (*p* = 0.130 by Delong’s test), but was significantly greater than the APACHE III score (*p* = 0.002 by Delong’s test, Fig. 6).

## Discussion

For the first time, this study employed a GA to develop a neural network model for the prediction of mortality in patients with ARDS. The model was validated in an external sample. The results showed that the most important predictors of mortality included immunodeficiency, metastatic tumor, hepatic failure, residing at home independently, FiO2, chronic dialysis, ventilation mode, albumin, age, highest glucose, highest bilirubin, minute ventilation volume and admit source. The model showed a significantly higher predictive performance than APACHE III scoring. Although the discrimination of the neural network model was higher than that developed by the logistic regression model, the difference was not statistically significant. In real clinical practice, the model can be used to stratify patients into risk subgroups. Furthermore, the variables used in the model were obtained within 24 h after ICU admission, which is fast enough to allow adequate time for interventions to take effect.

Mortality prediction in patients with ARDS has been extensively investigated in the literature. The APPS score incorporated the variables of age, plateau pressure and arterial oxygen partial pressure to fractional inspired oxygen ratio (PaO_2_/FiO_2_) (Villar et al., 2016). The score was a 9-point scale, in which a value greater than 7 had a mortality rate of 83.3% and a value below 5 had a mortality rate of 14.5% (*p* < 0.001). While the AUC was 0.755 in the original cohort, it was 0.62 in an independent cohort (Bos et al., 2016). Both peak and plateau inspiratory pressures were selected as important variables in predicting ARDS mortality in the stepwise regression model in the current study. Consistent with our findings, Panico et al. (2015) also showed that peak airway pressure (OR: 1.13; 95% CI [1.03–1.25]), rather than plateau pressure, was associated with mortality in a multivariable logistic regression model. Similar results were replicated in other studies (Erickson et al., 2007; Walkey & Wiener, 2011; Patel et al., 2016). Interestingly, Zhao and colleagues combined surfactant protein D (SP-D), interleukin-8, age and APACHE III score for the prediction of ARDS mortality, which reported a diagnostic performance comparable to our study. The addition of novel biomarkers significantly increases the predictive performance compared to a model incorporating simple clinical variables (Zhao et al., 2017). I proposed that the major drawback of the study was that the mechanical ventilation variables were not included. Since ARDS patients were primarily characterized by pulmonary dysfunctions, parameters of mechanical ventilation, such as peak inspiratory pressure, driving pressure and tidal volume must play an important role (Villar et al., 2017).

The patient type also plays an important role in determining ARDS mortality. In the present study, I found that living independently at home was associated with lower risks of mortality. Patients residing in an intermediate care facility had worse outcomes than those who resided independently at home, when they developed ARDS. This is not unique to ARDS, but had been reported in various conditions, such as ischemic colitis and pulmonary conditions (Peixoto et al., 2017; Duarte et al., 2017). However, most prediction models for ARDS failed to incorporate this factor (Frenzel et al., 2011; Zhang & Ni, 2015; Balzer et al., 2016; Luo et al., 2017), which might be responsible for their lack of satisfactory accuracy. Furthermore, the admission source (e.g., admit from operation room, emergency room, floor, stepdown unit or another hospital) was also an important predictor of mortality. There was evidence showing that patients admitted from the emergency room, had lower ventilator-associated lung injury than those admitted from other sources (Choudhuri, Chakravarty & Uppal, 2017). Furthermore, the mortality rates for overall ICU patients were quite different across various admission sources (Valentini et al., 2013). More recently, diffuse alveolar damage was found to be an important factor influencing clinical outcome (Kao et al., 2015; Cardinal-Fernández et al., 2017). However, this variable cannot be quantified routinely at the bedside, and thus the current analysis cannot include this variable. Perhaps the inclusion of this pathological variable can further improve the diagnostic performance of the predictive model.

Several limitations need to be acknowledged. Firstly, the study was retrospective and observational in design, which has inherent limitations, such as selection bias, loss to follow up and the presence of confounding factors. Further prospective studies are needed to evaluate the effectiveness of the prediction model in improving clinical outcomes. Secondly, the study employed only variables collected within the 24 h after ICU admission, failing to account for the dynamic process of disease progression. Dynamic indices have been shown to be superior to static indices in predicting clinical outcomes. In critical care medicine, such indices include stroke volume variation, lactate clearance rate and glucose variability (Pisarchik, Pochepen & Pisarchyk, 2012; Lee et al., 2015; Chao et al., 2017; Yi et al., 2017). It is not surprising that variables measured late in the disease course have better predictive performances than early ones. However, early predictions are more clinically useful than late ones, because the early prediction allows clinicians to have enough time to take action in order to reduce the mortality risk. It is a compromise between timeliness and accuracy, indicating that the improvement of accuracy is at the cost of delay. Thirdly, artificial intelligence and machine learning are suitable for prediction, but not necessarily for clinical decision making. Rather, there are many barriers to the implementation of ”black box” methods into the clinical workflow, which remains a relatively novel concept within medicine (Harrison et al., 2017). It is necessary to pilot prospective implementation studies of a tool based on this system in the critical care setting for patients with ARDS. This is a difficult task, however, lack of implementation research is a major limiting factor for models such as this one and the move from the realm of biomedical research to widespread use and application as clinical decision-making tools is challenging.

## Conclusion

In conclusion, the current study developed and validated a neural network model using GA for the prediction of mortality in patients with ARDS. The most important predictors of mortality were age, AIDS, leukemia, metastatic tumor, hepatic failure, highest bilirubin, and FiO2. The external validation of the model showed that the AUC was 0.821, which is greater than the APACHE III score and logistic regression model, albeit not statistically significant for the latter comparison.

##  Supplemental Information

10.7717/peerj.7719/supp-1 Supplemental Information 1 Candidate variables entered into genetic algorithm and their differences between the two cohorts

10.7717/peerj.7719/supp-2 Supplemental Information 2 code for analysis code for the performance of genetic algorithm and logistic regression model.

## Additional Information and Declarations

### Data Availability

The following information was supplied regarding data availability: The data are available at Biologic Specimen and Data Repository Information Coordinating Center: HLB01201414a.
https://biolincc.nhlbi.nih.gov/studies/sails/?q=acute%20respiratory%20distress.
