# Supplementary material for: Prediction model for patients with acute respiratory distress syndrome: use of a genetic algorithm to develop a neural network model
Source: PeerJ. 2019 Sep 16;7:e7719. doi: 10.7717/peerj.7719 (PMC6752189; doi:10.7717/peerj.7719)
Supplement: Supplemental Information 1 [file peerj-07-7719-s001.docx]

sTable 1. Candidate variables entered into genomic algorithm and their differences between the two cohort

| Variables | Details | Omega (n=272) | Sails (n=745) | p |
| --- | --- | --- | --- | --- |
| age (mean (sd)) | Age as appears on screening form (in years) | 54.31 (16.82) | 54.12 (16.34) | 0.869 |
| gender = 2 (%) | 1: male; 2: female | 133 ( 48.9) | 380 (51.0) | 0.600 |
| admtype (%) |  |  |  | 0.668 |
| 1 | Medical | 246 ( 90.4) | 655 (87.9) |  |
| 2 | Surgical scheduled | 8 ( 2.9) | 23 ( 3.1) |  |
| 3 | Surgical unscheduled | 15 ( 5.5) | 54 ( 7.2) |  |
| 4 | other | 3 ( 1.1) | 13 ( 1.7) |  |
| admitfrom (%) | Select the location where the patient was immediately prior to this ICU admission (OR, Recovery Room, ER, Floor, Another Special Care Unit, Another Hospital, Direct Admit, Step-down Unit). |  |  | 0.472 |
| 1 |  | 8 ( 2.9) | 24 ( 3.2) |  |
| 2 |  | 5 ( 1.8) | 8 ( 1.1) |  |
| 3 |  | 124 ( 45.6) | 291 (39.1) |  |
| 4 |  | 61 ( 22.4) | 210 (28.2) |  |
| 5 |  | 5 ( 1.8) | 17 ( 2.3) |  |
| 6 |  | 50 ( 18.4) | 152 (20.4) |  |
| 7 |  | 5 ( 1.8) | 11 ( 1.5) |  |
| 8 |  | 14 ( 5.1) | 32 ( 4.3) |  |
| reside (%) |  |  |  | 0.036 |
| 1 | Home independently | 220 ( 80.9) | 594 (79.8) |  |
| 2 | Home w/ help (supervision, direction, or personal assistance) | 35 ( 12.9) | 72 ( 9.7) |  |
| 3 | Home w/ professional help (nursing/nursing service) | 0 ( 0.0) | 14 ( 1.9) |  |
| 4 | Intermediate care or rehab facility | 3 ( 1.1) | 18 ( 2.4) |  |
| 5 | Skilled nursing facility | 8 ( 2.9) | 37 ( 5.0) |  |
| 7 | Others | 6 ( 2.2) | 9 ( 1.2) |  |
| surgel = 1 (%) | Is the patient  immediately postoperative  from  elective surgery? | 10 ( 3.7) | 19 ( 2.6) | 0.458 |
| icureadmit = 1 (%) | If item 8 is answered “yes”, was the readmission to the ICU within  24 hours of a previous ICU discharge? | 11 ( 4.0) | 29 ( 3.9) | 1.000 |
| chrondial = 1 (%) | Is the patient on  chronic dialysis or  peritoneal dialysis? | 10 ( 3.7) | 15 ( 2.0) | 0.198 |
| aids = 1 (%) | Select (Yes) or (No). Enter (No) if HIV positive but without other  AIDS criteria. | 8 ( 2.9) | 20 ( 2.7) | 0.996 |
| leuk = 1 (%) | Leukemia (AML,  CML, all lymphocytic  leukemia, multiple  myeloma) | 10 ( 3.7) | 43 ( 5.8) | 0.241 |
| lymph = 1 (%) | Non-Hodgkin’s  Lymphoma | 5 ( 1.8) | 10 ( 1.3) | 0.774 |
| tumor = 1 (%) | Solid Tumor with  metastasis | 2 ( 0.7) | 38 ( 5.1) | 0.003 |
| immune = 1 (%) | Select (Yes) or (No) to indicate if the patient is immunocompromised secondary to chemotherapy, radiation therapy, use of anti-rejection drugs taken after organ transplant, or the daily use of high doses of steroids (0.3 mg Prednisone kg/day or equivalent therapy) within part of or the entire previous six months. | 25 ( 9.2) | 118 (15.8) | 0.009 |
| hepa = 1 (%) | Select (Yes) or (No) to indicate if the patient has  decompensated cirrhosis (Hepatic Failure) as evidenced by  one or more episodes of jaundice and ascites, upper  gastrointestinal bleeding or hepatic encephalopathy or  comas. | 1 ( 0.4) | 11 ( 1.5) | 0.262 |
| cirr = 1 (%) | Select "1" (Yes) or "2" (No) to indicate if the patient has  cirrhosis without the stigmata indicated above in 17. If the  patient has a functioning liver transplant, this chronic health  item would not apply. | 12 ( 4.4) | 38 ( 5.1) | 0.772 |
| diab = 1 (%) | Diabetes Mellitus | 88 ( 32.4) | 170 (22.8) | 0.003 |
| hyper = 1 (%) | Hypertension | 131 ( 48.2) | 356 (47.8) | 0.986 |
| myocard = 1 (%) | Prior myocardial  infarction | 23 ( 8.5) | 29 ( 3.9) | 0.006 |
| heart = 1 (%) | Chronic heart failure | 19 ( 7.0) | 48 ( 6.5) | 0.872 |
| vascular = 1 (%) | Peripheral vascular  disease | 12 ( 4.4) | 36 ( 4.8) | 0.910 |
| aestroke = 1 (%) | Prior stroke with  sequelae | 10 ( 3.7) | 20 ( 2.7) | 0.536 |
| dementia = 1 (%) | Dementia | 10 ( 3.7) | 27 ( 3.6) | 1.000 |
| chrpulm = 1 (%) | Chronic pulmonary  disease | 36 ( 13.2) | 130 (17.5) | 0.128 |
| arthritis = 1 (%) | Arthritis | 19 ( 7.0) | 73 ( 9.8) | 0.205 |
| ulcer = 1 (%) | Peptic ulcer disease | 8 ( 2.9) | 53 ( 7.1) | 0.020 |
| vasol24 = 1 (%) | Select (Yes) or (No) to indicate if the pt has received any  vasopressors in the 24 hours prior to randomization. | 140 ( 51.5) | 406 (54.6) | 0.420 |
| templ (mean (sd)) | Enter the lowest temperatures in Centigrade or Fahrenheit. Add 1 degree Centigrade or 2 degrees  Fahrenheit if axillary temperatures. | 36.50 (1.07) | 36.56 (0.88) | 0.354 |
| temph (mean (sd)) | Enter the highest temperatures in Centigrade or Fahrenheit. Add 1 degree Centigrade or 2 degrees  Fahrenheit if axillary temperatures. | 38.12 (1.19) | 38.09 (0.96) | 0.639 |
| sysbpl (mean (sd)) | The lowest systolic blood pressure | 86.34 (17.34) | 85.38 (15.62) | 0.399 |
| sysbph (mean (sd)) | The highest systolic blood pressure | 145.08 (25.77) | 144.01 (25.79) | 0.559 |
| mapl (mean (sd)) | Lowest mean arterial pressure | 59.49 (12.95) | 59.95 (11.27) | 0.585 |
| maph (mean (sd)) | highest mean arterial pressure | 96.24 (17.89) | 98.27 (17.96) | 0.113 |
| hratel (mean (sd)) | Low heart rate | 85.08 (20.13) | 83.19 (18.11) | 0.152 |
| hrateh (mean (sd)) | High heart rate | 117.29 (20.95) | 118.37 (22.74) | 0.497 |
| respl (mean (sd)) | Low respiratory rate | 18.15 (5.98) | 17.42 (6.25) | 0.096 |
| resph (mean (sd)) | High respiratory rate | 32.91 (8.88) | 32.92 (8.50) | 0.994 |
| ventl = 1 (%) | Was patient ventilated when the lowest  respiratory rate occurred? | 247 ( 90.8) | 689 (92.5) | 0.458 |
| venth = 1 (%) | Was patient ventilated when the highest  respiratory rate occurred? | 237 ( 87.1) | 635 (85.2) | 0.506 |
| urineout_0 (mean (sd)) | Enter the amount of urine output (ml) in the 24 hrs prior to randomization time. E.g., if time of randomization occurs  on 2/1/07 at 1400, then the urinary output listed should be from 1/31/07 at 1400 to 2/1/07 at 1400). If a large volume  of urine was inadvertently spilled or the urine was not measured, mark the field as “unknown”. A urine output value of zero indicates that data are available and the  patient produced no urine. | 1655.99 (1262.99) | 1604.72 (1236.42) | 0.562 |
| fluidout_0 (mean (sd)) | Enter the total fluid intake (ml) in the 24 hrs prior to randomization. (See example in number 8). This total should INCLUDE urine output and a negative CVVH balance. | 2054.25 (2223.36) | 1880.83 (1394.46) | 0.141 |
| fluidin_0 (mean (sd)) | Enter the total fluid intake (ml) in the 24 hrs prior to randomization. (See example in number 8). This total should include a positive CVVH balance. | 4752.95 (3335.14) | 4057.34 (2617.34) | 0.001 |
| hctl (mean (sd)) | Enter lowest % values rounded to the nearest whole number (e.g., "35", not " .35"). If only one value is present for 24-hour period, enter this value in the “only column”. | 29.55 (5.92) | 29.94 (6.14) | 0.360 |
| hcth (mean (sd)) | Enter highest % values rounded to the nearest whole number (e.g., "35", not " .35"). If only one value is present for 24-hour period, enter this value in the “only column”. | 31.65 (6.14) | 31.85 (6.54) | 0.658 |
| wbcl (mean (sd)) | Enter lowest as "00000" (e.g., a WBC of 14.2 should be entered as "14200").  If only one value is present for 24-hour period, enter this value in the “only column”. | 11958.65 (9705.70) | 14148.16 (12238.23) | 0.008 |
| wbch (mean (sd)) | Enter highest as "00000" (e.g., a WBC of 14.2 should be entered as "14200").  If only one value is present for 24-hour period, enter this value in the “only column”. | 13773.73 (9906.71) | 15738.45 (12368.95) | 0.019 |
| plate (mean (sd)) | Enter only the lowest value during the 24 hours. Enter as "000" (e.g., a platelet count of 258,000 should be entered as "258"). | 192.55 (113.22) | 186.25 (124.63) | 0.467 |
| sodiuml (mean (sd)) | Enter lowest.  If only one value present for 24-hour period, enter this value in the “only column”. | 137.52 (5.22) | 137.79 (5.43) | 0.468 |
| sodiumh (mean (sd)) | Enter highest.  If only one value present for 24-hour period, enter this value in the “only column”. | 139.71 (5.03) | 139.60 (5.27) | 0.769 |
| potasl (mean (sd)) | Enter highest and lowest.  If only one value present for 24-hour period, enter this value in the “only column”. | 3.82 (0.64) | 3.84 (0.62) | 0.672 |
| potash (mean (sd)) | Enter highest and lowest.  If only one value present for 24-hour period, enter this value in the “only column”. | 4.30 (0.82) | 4.23 (0.72) | 0.186 |
| bun (mean (sd)) | Enter only highest value. | 30.64 (23.79) | 27.56 (20.26) | 0.041 |
| creatl (mean (sd)) | Enter highest and lowest. If only one value present for 24-hour period, enter this value in the “only column”.  If only one value present for 24-hour period, enter this value in the “only column”. | 1.68 (1.58) | 1.37 (1.08) | <0.001 |
| creath (mean (sd)) | Enter highest and lowest. If only one value present for 24-hour period, enter this value in the “only column”.  If only one value present for 24-hour period, enter this value in the “only column”. | 1.91 (1.75) | 1.53 (1.18) | <0.001 |
| glucl (mean (sd)) | Enter highest and lowest. If only one value present for 24-hour period, enter this value in the “only column”.  If only one value present for 24-hour period, enter this value in the “only column”. | 113.53 (61.06) | 124.77 (48.78) | 0.003 |
| gluch (mean (sd)) | Enter highest and lowest. If only one value present for 24-hour period, enter this value in the “only column”.  If only one value present for 24-hour period, enter this value in the “only column”. | 165.60 (81.87) | 167.08 (86.72) | 0.808 |
| albuml (mean (sd)) | Enter highest and lowest. If only one value present for 24-hour period, enter this value in the “only column”.  If only one value present for 24-hour period, enter this value in the “only column”. | 2.28 (0.65) | 2.19 (0.64) | 0.063 |
| albumh (mean (sd)) | Enter highest and lowest. If only one value present for 24-hour period, enter this value in the “only column”.  If only one value present for 24-hour period, enter this value in the “only column”. | 2.39 (0.76) | 2.27 (0.68) | 0.032 |
| bilih (mean (sd)) | Enter only highest value. | 1.64 (2.79) | 1.32 (1.80) | 0.044 |
| bicarbl (mean (sd)) | Enter only lowest value. | 21.08 (4.92) | 21.78 (5.52) | 0.067 |
| ck_0 (mean (sd)) | Potassium | NaN (NA) | 219.20 (406.96) | NA |
| alt_0 (mean (sd)) | ALT | NaN (NA) | 36.30 (35.35) | NA |
| ast (mean (sd)) | AST | NaN (NA) | 51.86 (57.93) | NA |
| crp_0 (mean (sd)) | CRP | NaN (NA) | 26.44 (31.10) | NA |
| simv_0 = NaN (%) | Ventilator mode (NaN indicates the mode was not used) | 262 ( 96.3) | 0 ( 0.0) | <0.001 |
| prvc_0 = NaN (%) | Ventilator mode (NaN indicates the mode was not used) | 229 ( 84.2) | 0 ( 0.0) | <0.001 |
| pressup_0 = NaN (%) | Ventilator mode (NaN indicates the mode was not used) | 249 ( 91.5) | 0 ( 0.0) | <0.001 |
| volassist_0 = NaN (%) | Ventilator mode (NaN indicates the mode was not used) | 87 ( 32.0) | 0 ( 0.0) | <0.001 |
| pcirv_0 = NaN (%) | Ventilator mode (NaN indicates the mode was not used) | 271 ( 99.6) | 0 ( 0.0) | <0.001 |
| aprv_0 = NaN (%) | Ventilator mode (NaN indicates the mode was not used) | 264 ( 97.1) | 0 ( 0.0) | <0.001 |
| ventoth_0 = NaN (%) | Ventilator mode (NaN indicates the mode was not used) | 268 ( 98.5) | 0 ( 0.0) | <0.001 |
| hfov_0 = NaN (%) | Ventilator mode (NaN indicates the mode was not used) | 272 (100.0) | 0 ( 0.0) | <0.001 |
| tidal_0 (mean (sd)) | Enter the corrected inspired tidal volume: inspired tidal volume (ml) set on the ventilator minus any additional tidal volume added to correct for has compression and ventilator tube expansion (this should = the tidal volume called for by the protocol; this will not = the volume set on the ventilator unless the ventilator makes automatic adjustments for gas compression/tube expansion).  Puritan-Bennett 7200’s and some other ventilators make this correction automatically (for these vents, the value set on the vent = the calculated delivered tidal volume). | 428.50 (89.31) | 413.46 (87.01) | 0.025 |
| setrate_0 (mean (sd)) | Enter the rate set on the ventilator if the patient is on SIMV, SIMV with Pressure Support, Assist/Control, or Pressure Control mode. (This is the minimum rate set on the ventilator, not the patient rate). | 21.09 (7.39) | 21.96 (7.32) | 0.112 |
| resp_0 (mean (sd)) | Enter the total respiratory rate, which may exceed the Set Rate above if the patient is making additional inspiratory efforts. | 25.21 (7.18) | 25.09 (7.15) | 0.814 |
| minvent_0 (mean (sd)) | Enter the total minute ventilation in liters per minute. This value is available from a digital report on the ventilator. | 10.99 (3.03) | 10.77 (3.15) | 0.346 |
| peep_0 (mean (sd)) | Enter the PEEP applied on the ventilator in cmH2O.  This is the external or applied PEEP, not the total PEEP, auto-PEEP, or intrinsic PEEP. | 8.61 (3.41) | 9.20 (3.80) | 0.024 |
| fio2_0 (mean (sd)) | Enter FiO2 prior to randomization. | 0.56 (0.18) | 0.56 (0.19) | 0.614 |
| SpO2_0 (mean (sd)) | Enter SpO2 prior to randomization. | 95.68 (3.69) | 95.98 (3.51) | 0.234 |
| pplat_0 (mean (sd)) | Enter the value for plateau pressure measurement in cm H20.  The plateau pressure measurement should be made with a 0.5 second inspiratory pause. | 23.06 (5.19) | 23.49 (6.60) | 0.422 |
| pip_0 (mean (sd)) | Enter the peak inspiratory airway pressure (cmH2O).  This should be obtained while the patient is relaxed, not coughing or moving in bed. | 27.60 (8.40) | 27.28 (8.56) | 0.608 |
| meanair_0 (mean (sd)) | Enter the mean airway pressure (cmH2O).  This should be obtained while the patient is relaxed, not coughing or moving in bed. | 14.19 (4.35) | 14.99 (4.91) | 0.024 |
| status (%) |  |  |  | 0.172 |
| 1 | Select “home with UAB” if the patient is home with unassisted  breathing at any time up through day 90. “Home” is defined as the place the patient lived prior to study hospital admission (i.e., pt living in a nursing home→admitted to study hospital and enrolled into study→DC’d back to nursing home on UAB. The nursing home would qualify as “home on UAB”. Pts previously living at home who are discharged to a rehab facility on UAB from study hospital would NOT qualify as being “home on UAB”.) | 196 ( 72.1) | 497 (66.7) |  |
| 2 | Select “Dead…” if the patient died prior to discharge home with unassisted breathing or died prior to achieving unassisted breathing at home for 48 hours. | 59 ( 21.7) | 205 (27.5) |  |
| 3 | Select “Other” if neither condition above applies. E.g., if the patient went home on assisted breathing and has not achieved unassisted breathing for 48 hours, continues on assisted breathing, or has been transferred to another facility, other than home, on unassisted breathing. | 17 ( 6.2) | 43 ( 5.8) |  |
